# Comment: Comparison of Large-Bore Thrombectomy With Catheter-Directed Thrombolysis for the Treatment of Pulmonary Embolism

**DOI:** 10.1016/j.jscai.2023.100613

**Published:** 2023-03-16

**Authors:** Josip A. Borovac, Dino Miric, Mislav Lozo, Anteo Bradaric Slujo, Jaksa Zanchi

**Affiliations:** a Division of Interventional Cardiology, Cardiovascular Diseases Department, University Hospital of Split, Split, Croatia; b Department of Pathophysiology, University of Split School of Medicine, Split, Croatia

**Keywords:** catheter-directed therapies, pulmonary embolism, venous access

Feroze et al^1^ retrospectively investigated important problem in contemporary pulmonary embolism (PE) practice by demonstrating that there was no difference in mortality and rehospitalization rates in patients with intermediate-to-high-risk (IHR) PE treated with large-bore thrombectomy compared with patients treated with catheter-directed thrombolysis (CDT). Furthermore, both treatment approaches for PE were similar concerning safety.

Given the scarcity of randomized data in this setting and inconclusive evidence base regarding hard clinical end points,^2^^,^^3^ we would wish to complement the authors' findings with our own recently published retrospective data.^4^

In our cohort of patients presenting with IHR acute PE, we performed CDT through the superficial cubital vein using a standard pigtail catheter and administering alteplase locally. This method had a robust effect on hemodynamics because it significantly reduced systolic pulmonary artery pressure, and mean pulmonary artery pressure, and improved mean systemic perfusion pressure at 12 hours after intervention with a concomitant reduction in the Miller score and index, both reflecting the angiographic severity of pulmonary artery obstruction and perfusion. Of importance, the rates of adverse events, particularly bleeding, were low with 2 bleeding events of which only 1 required the transfusion of 1 unit of packed red blood cells. Furthermore, procedural success was 100% with no intracranial hemorrhage, access-site bleeding, or death events. However, no comparator was available in our study.

Taken together, data from the study by Feroze et al^1^ and our own suggest that CDT in patients with IHR acute PE is complementary to mechanical thrombectomy and is equally safe and effective procedure that can be successfully performed by cannulating either small or large veins by experienced operators. This is exquisitely important from the pharmacoeconomic standpoint. An option to use readily available catheters outside of dedicated thrombolytic or thrombectomy systems may provide significant economical savings, especially in resource-limited health care systems. Finally, both interventional approaches have a great potential to help a large proportion of patients with PE, and a transcubital access is also a feasible option in this scenario. However, as Feroze et al^1^ rightly conclude, adequately powered randomized studies that would examine percutaneous thrombectomy versus CDT and optimal medical therapy are needed to confirm the clear signal of benefit associated with catheter modalities. Finally, these observations should be put in the context with the recently published consensus statement on percutaneous therapies in acute PE, stipulating that CDT-based strategies should be considered in all cases of IHR acute PE in whom systemic thrombolysis is contraindicated or failed or there was a hemodynamic deterioration despite full therapeutic anticoagulation.^5^
